# Systemic inflammation and the risk of depression in people with type 2 diabetes

**DOI:** 10.1093/eurpub/ckac129.701

**Published:** 2022-10-25

**Authors:** N Schmitz, E Graham, S Deschenes

**Affiliations:** 1 Tuebingen University, Population-Based Medicine, Tuebingen, Germany; 2 McGill University, Psychiatry & Epidemiology, Montreal, Canada; 3 University College Dublin, Psychology, Dublin, Ireland

## Abstract

**Background:**

Depression is a common co-morbidity in diabetes. The mechanisms underlying the association between depression and diabetes are poorly understood. Although risk factors, such as poor lifestyle behaviours, obesity, and stress have been identified, emerging evidence suggests that systemic inflammation may play an important role in the pathogenesis and recurrence of depression in people with diabetes. The aim of the present study was to evaluate if the inflammatory marker C-reactive protein (CRP) is associated with an increased risk of major depression episodes in people with type 2 diabetes.

**Methods:**

A prospective, community-based study was conducted in Quebec, Canada. Individuals were recruited from the CARTaGENE (CaG) cohort, a population-based survey of Quebec residents aged 40 to 69 years. Our sample included 719 individuals with type 2 diabetes and 1423 individuals without diabetes. Individuals were assessed at baseline and 5 years after baseline. Major depression disorders were assessed using a clinical interview (CIDI). Inflammatory markers were assessed from blood samples. Elevated CRP levels were defined as ≥ 3 mg/L.

**Results:**

Participants with both diabetes and elevated CRP levels had the highest risk of major depressive episodes (adjusted OR = 1.90, 95% CI 1.45, 2.50), compared to those without diabetes and without elevated CRP levels. The risk of major depressive episodes in individuals with diabetes without elevated CRP episodes was lower (adjusted OR = 1.21, 95% CI 0.85, 1.73) and similar to the risk of those without diabetes and elevated CRP levels (adjusted OR = 1.15, 95% CI 0.94, 1.39).

**Discussion:**

The study highlights the interaction between diabetes, inflammatory makers, and depression in a community sample. Early identification, monitoring, and management of elevated inflammation levels might be an important depression prevention strategy in people with type 2 diabetes.

